# Lung cancer screening: the way forward

**DOI:** 10.1038/sj.bjc.6604509

**Published:** 2008-07-29

**Authors:** J K Field, S W Duffy

**Affiliations:** 1 Department of Surgery and Oncology, Roy Castle Lung Cancer Research Programme, University of Liverpool Cancer Research Centre, Liverpool L3 9TA, UK; 2 Cancer Research UK Centre for Epidemiology, Mathematics and Statistics, Wolfson Institute of Preventive Medicine, Charterhouse Square, London EC1M 6BQ, UK

**Keywords:** lung cancer, screening, chest X-ray, CT screening, cost-effectiveness, risk modelling

## Abstract

To take lung cancer screening into national programmes, we first have to answer the question whether low-dose computed tomography (LDCT) screening and treatment of early lesions will decrease lung cancer mortality compared with a control group, to accurately estimate the balance of benefits and harms, and to determine the cost-effectiveness of the intervention.

Lung cancer kills more people worldwide than other malignancy. The number of deaths in the western world has fallen in the past years and this is likely to be due to a greater public awareness as well as successes in smoking cessation programmes. Unfortunately, the tobacco epidemic is still growing in Southeast Asia and China as the tobacco industry has now concentrated its sales in these regions. However, there is now a large ex-smoking population in the United States and Europe, who remain at a very high risk of developing lung cancer, which is dependent on their smoking duration before tobacco cessation. This group of individuals now exceeds current smokers in both the United States and Europe and will continue to do so over the next two to three decades. National health-care programmes would have a responsibility, if there were a proven screening tool, to provide a mechanism by which these high-risk individuals are identified and targeted for lung cancer screening. Screening must be instigated before patients develop any symptoms, as surgical resection at an early stage of the disease remains the only realistic option for a cure.

## Chest X-ray and sputum cytology lung cancer screening

The earliest lung screening trial was undertaken in London with over 55 000 individuals randomised to chest X-ray every 6 months for 3 years or chest X-ray at the beginning and end of the 3-year period (Brett, 1969). No mortality difference was found between the two groups. Three major trials in the United States and one in Czechoslovakia were developed in the 1970s, as outlined in Table 1. The results of these large trials were disappointing as none of these studies showed any reduction in lung cancer mortality utilising chest X-ray, with or without, sputum cytology. However, some design features of these trials have been criticised on the basis of active early detection measures in the control arm in many of the studies, possible suboptimal selection of study populations, and of arguably inadequate sample sizes (Prorok *et al*, 2000). Many of these criticisms have now been taken on board by the current lung cancer screening trials.

One current trial, which has ‘usual care’ only in the control arm, is the lung component of the NCI PLCO (Prostate, Lung Colorectal and Ovarian) screening trial. In this trial, smokers are offered annual chest X-ray for 3 years, and non-smokers two annual repeat screens; the results of this study are not expected until 2010.

## Low-dose computed tomography lung cancer screening: observational studies

Low-dose computed tomography (LDCT) offers a major advance in imaging technology, which was first introduced in the 1990s (Naidich *et al*, 1990) and later by Reeves and Kostis (2000). This is more sensitive than chest X-ray and has enabled the detection of lung tumours less than 1 cm; thus, allowing a complete scan on the thorax in less than 30 s. Randomised trials of this technology as a screening tool have not as yet been completed, but there have been a number of demonstration projects (Table 2). Early studies of note include the Early Lung Cancer Action Project (ELCAP) (Henschke *et al*, 1999) in 1000 high-risk smokers; the Mayo Clinic Project with 1520 individuals aged 50 years having annual sputum cytology and spiral CT screening (Swensen *et al*, 2000); and a 3-year mass screening programme using a mobile CT unit in Japan (Sone *et al*, 1998).

The ELCAP study enroled 1000 symptom-free individuals aged 60 years or more with >10 pack-years history of smoking, who were fit to undergo surgery into a study. All individuals underwent an annual spiral CT and chest X-ray. The lung cancer detection rate was 2.7% in the first year and 0.7% in the second year (incidence), and this study also demonstrated that the sensitivity of low-dose spiral CT for early lung cancer was far greater than for chest X-rays. The majority of ‘screen-detected’ tumours were at an early stage and suitable for surgery. This seminal paper by Henschke and co-workers (Henschke *et al*, 1999) re-ignited interest and debate in developing new lung cancer screening trials in the United States and Europe. Other demonstration projects found similar results (Table 2). The Early Lung Cancer Action Project has since been expanded to a major international collaboration, I-ELCAP, with more than 30 000 screenees (see below), with similar findings to the original New York project (Henschke *et al*, 2006). The authors also estimated a very high case survival rate for stage I tumours undergoing surgery. There is, however, considerable debate around the interpretation of increased survival in LDCT-diagnosed cancers, as longer survival does not necessarily equate to reduced mortality (Twombly, 2007). In addition to concerns about self-selection for surgery (or for no surgery) among stage 1 patients, the major reservation relates to overdiagnosis of tumours, which would not have been life threatening and would never have come to clinical attention in the absence of screening. The previous generation of chest X-ray trials suggested a measure of overdiagnosis (Kubik *et al*, 2000; Marcus *et al*, 2000). The much greater sensitivity of LDCT has, in turn, led to fears of an increased risk of overdiagnosis. The most balanced arguments to date concerning the IELCAP findings have been in a recent *BMJ* editorial (McMahon and Christiani, 2007). The authors' view is that the objective of lung cancer screening is to reduce lung cancer mortality, and it is not possible to confidently conclude this from the IELCAP study.

The one other large observational analysis is by Bach *et al* (2007) whose conclusions were diametrically opposed to those from by the IELCAP Consortium. Bach and colleagues used data from 3246 current or former smokers who entered into screening studies in the United States and in Italy, with follow-up for a median of 3.9 years. They used a model of predicted risk of lung cancer mortality to estimate the expected numbers of lung cancer deaths and compared these with the corresponding observed deaths; they found no decrease in the number of diagnoses of advanced lung cancers or deaths from lung cancer (38 deaths due to lung cancer observed and 38.8 expected; RR 1.0; 95% CI: 0.7–1.3; *P*=0.90). The authors concluded that there was no evidence of a mortality advantage with LDCT screening from this study. However, their exclusion of deaths from tumours diagnosed early in the period of observation has been criticised, as have been various other assumptions and procedures in their approach.

## LDCT lung cancer screening: randomised trials

The EU-US spiral CT Collaboration was initiated in 2001 in Liverpool. Subsequent meetings throughout Europe resulted in the development of collaborative protocols on radiology, pathology, minimum datasets, treatment, as well as core LDCT protocol. This provided a mechanism by which the different trial groups could work together with the ultimate aim to pool their data, thereby enhancing the overall power of these studies and potentially reporting earlier; the concept of which was formulated in the ‘Liverpool Statement 2005’ (Field *et al*, 2006).

The randomised trials of LDCT are summarised in Table 3. The first major RCT lung cancer screening trial utilising LDCT was the National Lung cancer Screening Trial (NLST), which is a combination of two trials, one set up by the US National Cancer Institute (NCI) and the other by the American College of Radiology Imaging Network (ACRIN). The NLST started in 2002 and completed enroling in 2004. This study has over 50 000 former and current smokers randomised to annual LDCT or annual chest X-ray for 3 years. The major objective of this was to determine whether LDCT reduces lung cancer mortality compared with a chest X-ray arm. (http://www.cancer.gov/NLST). This trial will be completed in 2009 and aims to report in 2012; it is designed to have a 90% power to detect a mortality reduction of 20%.

The NELSON trial was launched in 2003 in the Netherlands and Belgium (van Iersel *et al*, 2007), and now incorporates centres in Denmark. This trial is designed to compare lung cancer mortality in a group randomised to LDCT screening with a control group, without screening. This trial aims to report in 2014 and with 20 000 recruits and is designed to have a power of 80%, significance level of 0.05 to detect a mortality reduction of 20%; a 95% compliance in the screen group, a 5% contamination rate in the control group and 10 years follow-up after randomisation. A great deal of attention was focused on the selection of patients for NELSON in order to focus on the highest risk groups and thus reduce the cost of the RCT but retain the power of the study. Potential study participants were approached by letter with a questionnaire on their smoking exposure and whether they wished to be included in the trial. The questionnaire was initially sent to 335 441 men and women aged 50–75 years old. On the basis of this data set the selection criteria were developed, depending on the duration of smoking, duration of smoking cessation in ex-smokers, number of cigarettes smoked per day, and the mean estimated expected lung cancer mortality rate. In this trial, LDCT screening takes place in years 1, 2, and 4, with 10 years of follow-up. The trial has 20 000 individuals randomised in equal numbers to LDCT or ‘usual care’.

A number of small trials have been initiated in anticipation of combination with partner studies or a future meta-analysis. These include the *ItaLung* and *Dante* Trials in Italy (Picozzi *et al*, 2005; Infante *et al*, 2007).

The French randomised pilot study, Depiscan, comparing LDCT and chest X-ray recently reported its baseline findings (Blanchon *et al*, 2007); in this the selection of participants was undertaken by General Practioners (GPs) and occupational physicians. Eligible subjects were males and females aged 50–75 years with either a current or former smoking history of at least 15 cigarettes per day for 20 years. The screening was undertaken annually for 2 years. The objective was to enrol 1000 subjects; 765 have been recruited with 621of these having complete imaging baseline data. Non-compliance was an important issue in this study and the recruitment took twice as long as envisaged. Eight lung cancers were detected in the LDCT arm (2.4%) and one (<1%) in the chest X-ray arm.

## National lung cancer screening programme

To date, we do not have the results of any randomised trials, which can provide adequate evidence to justify the instigation of a National Lung Cancer Screening Programme. The results of the NLST and NELSON studies are eagerly awaited. The unanswered question that remains in the United Kingdom is whether either of these studies will provide adequate information on their own to justify the implementation of a UK National Screening Programme? Although the combined US study is large and should have precise results, the use of an active screening regime in the control group may raise problems of interpretation. The NELSON study has adequate power for a substantial benefit in a high-risk group, but a lower baseline lung cancer mortality or smaller benefit than anticipated may jeopardise a conclusive result.

The UK National Screening Committee has determined 22 criteria for the viability, effectiveness, and appropriateness of a screening programme (http://www.nsc.nhs.uk/uk_nsc/uk_nsc_ind.htm); 20 of which are relevant to LDCT lung cancer screening. Black *et al* (2007) have undertaken a systematic review of the literature to ascertain whether there was evidence for any clinical effectiveness utilising LDCT for lung cancer screening. This extremely detailed review was undertaken at the time when there was a paucity of real data, and thus their conclusions were drawn from two small trials with very variable results. Not surprisingly, their conclusion stated that there was insufficient evidence at the time to support LDCT screening.

The current lack of evidence and the possibility of inconclusive results from relatively small group of current trials would suggest that a UK trial would make a valuable contribution to the research effort worldwide and answer questions particularly pertinent to the UK health environment. It is a salutary fact that four decades after the development of this ‘technology’, we still do not have experimental evidence for or against the implementation of this screening modality. Lung cancer kills more individuals in the United Kingdom than any other malignancy. Our responsibility is not only to determine whether LDCT screening and treatment of early lesions will decrease lung cancer mortality compared with a control group without screening but also to test this against the criteria outlined by the UK Screening Committee, especially those concerning cost-effectiveness. A useful aid to cost-effectiveness is the ability to select a population at sufficiently high risk to give a substantial harvest of tumours in return for the screening activity. The Liverpool Lung Project Risk Model provides an opportunity for this (Field *et al*, 2007; Cassidy *et al*, 2008). The risk groups selected are those for whom the benefits of the screening will outweigh the likely harms.

The cost-effectiveness of lung cancer LDCT screening has been estimated by a number of groups, which were reviewed by Black *et al* (2006), who found the current estimates difficult to interpret and certainly not definitive. In response to a request from the UK National Cancer Research Institute, Whynes (2008) developed a simple and transparent economic model based on UK costings and the empirical clinical data are currently available. The UK cost-effectiveness model used a simple, deterministic approach to the modelling of a screening regimen. The model required only a limited number of parameters. The expected mortality gain as a result of screening was estimated by combining published survival data from screened and unscreened cohorts with routinely published national mortality figures. Conservative costs were estimated where there was uncertainty over any specific parameter, thus probably resulting in less cost-effective screening. The incremental cost-effectiveness ratio of a single CT screen among a high-risk male population was calculated to be around £14 000 per quality-adjusted life year gained, if the anticipated mortality benefit was indeed observed. Sensitivity analysis was carried out with a range of differing assumptions, providing a range of cost-effectiveness ratios as high as £21 000 or as low as around £6000. In the United Kingdom, the National Institute and Clinical Excellence (NICE) evaluated both clinical and cost-effectiveness when deciding on recommendations to implement new interventions. Currently, NICE considered ICERs below £20 000 per QUALY as definitely acceptable and costs up to £30 000 as suitable for consideration.

The approval of any future lung cancer screening trial will evidently be dependent on costings in line with current political health economics; however, this defining factor was not applicable for either breast cancer screening, which was set up after the Forest Report in 1985 (Gerard *et al*, 1997), or cervical cancer screening, which was set up in 1992 (Quinn *et al*, 1999). The most efficient way of controlling cost, however, will be to screen those individuals who are at high risk of developing the disease. There has been increasing interest in developing methods for individual risk prediction for lung cancer. Models have been developed for use within high-risk groups (Bach *et al*, 2003), and for the general population (van Klaveren *et al*, 2002), which rely only on age and smoking. Epidemiological risk factors usually show poor discrimination between those ‘who do’ and ‘do not’ develop disease (Wald *et al*, 1999), but lung cancer is an exception, in that a high proportion of cases are attributable to one risk factor, smoking. The predictive accuracy of lung cancer risk models may be further improved by the addition of other epidemiological risk factors, including smoking history variables, environmental tobacco smoke, family history of cancer, prior respiratory disease, and occupational exposures (dust and asbestos) (Cassidy *et al*, 2007, 2008; Spitz *et al*, 2007). The Liverpool Lung Project (LLP) (Field *et al*, 2005) has recently developed a method to calculate absolute risk of lung cancer over a defined period, based on data from a case–control study of lung cancer in Liverpool. Significant risk factors in the final model were smoking duration, family history of lung cancer, history of non-pulmonary malignant tumour, history of pneumonia, and occupational exposure to asbestos. These factors were combined with published age- and sex-specific incidence rates to give absolute probability of lung cancer development within 5 years. In comparison with previous lung cancer prediction models, the LLP risk model has distinctive strengths. First, the predictor variables are all explicitly defined and can be readily assessed at the time of patient presentation, and secondly, patients can be assigned to their appropriate risk class on the basis of information from the initial history alone. The LLP Risk Model requires rigorous validation in a separate population.

## Conclusion

Currently, the treatment of advanced lung cancer is inadequate and, thus, there is an urgent pressure to implement screening programmes in many countries. In the United Kingdom, no decision will be made until we have the results of the current international RCT trials and, hopefully, those from a future UK lung cancer screening RCT. However, time is not on our side with over 32 000 individuals a year dying from lung cancer in the United Kingdom, and this statistic alone should accelerate progress in reaching a conclusion concerning the feasibility of lung cancer screening.

## Figures and Tables

**Table 1:** Chest X-ray +/− sputum cytology lung cancer screening trials

**Lung cancer screening trial**	**Trial design**	**No. of participants**	**Lung cancer detected**	**Lung cancer mortality^a^**	**Reference**
London	Chest X-ray, 6 months, 3 years	29 723	132	2.1	Brett (1969)
	*vs* chest X-ray only at the end of year 3	25 311	96	2.4	
					
MSKCC Lung Cancer Screening	A chest X-ray and sputum cytology, every 4 months	49 68	144	2.7	Melamed *et al* (1984)
Programme	*vs* chest X-ray annually	5072	144	2.7	
Johns Hopkins Lung Project	A chest X-ray and sputum cytology every 4 months	5226	202	3.4	Frost *et al* (1984)
	*vs* chest X-ray annually	5161	206	3.8	
Mayo Lung Project	Chest X-ray and sputum cytology every 4 months, 6 years	4618	160	3.9	Marcus *et al* (2000)
	*vs* chest X-ray and sputum cytology annually	4593	39	3.6	
Czechoslovakia	Chest X-ray and sputum cytology every 6 months, 3 years	3172	39	3.6	Kubik *et al* (2000)
	*vs* chest X-ray and sputum cytology beginning of first year and the end of third year	3174	27	2.6	
					
PLCO	Lung aspect of trial: chest X-ray: smokers had test at the entry and annually for 3 years. Never smokers had the test at the entry and annually for 2 years	77 469	Not published		Gohagan *et al* (2000)
	*vs* usual care	77 468			

PLCO=Prostate, Lung Colorectal and Ovarian.

a Per 1000 person-years.

**Table 2:** LDCT lung cancer screening in observational studies

**Reference**	**No. of participants**	**Smokers (PKS)**	**No. of non-calcified nodules**	**No. of lung cancers**
Henschke (2000)	1000	Smokers	233	27
		PYS >10	63 (incidence)	7 (incidence)
		Asbestos 14%		
				
Sone *et al* (1998)	5483	Smokers ∼50%	279	22
		PYS >1		
				
Swensen *et al* (2002)	1520	Smokers	2244	25
		PYS >20	588 (incidence)	10(incidence)
				
Sobue *et al* (2002)	1611	Smokers ∼85%	186	14
		PYS 50%	721 (incidence)	8 (incidence)
				
Tiitola *et al* (2002)	602	Smokers >95%	111	5
		PYS>10		
		Asbestos 100%		
				
Nawa *et al* (2002)	7956	Smokers >60%	2865	41
		PYS=50%		
				
Diederich *et al* (2002)	817	Smokers 100%	858	12
		PYS >20	174 (incidence)	10 (incidence)
MacRedmond *et al* (2004)	449	Smokers 100%	155	2
		PYS >10		
		Asbestos 7.6%		
				
Stephenson *et al* (2005)	87	Smokers 100%		4
		PYS >20		
				
Chong *et al* (2005)	6406	Smokers 100%	2255	11
		PYS >20		
				
Henschke *et al* (2006)	31567	Smokers	ND	484
		PYS 15–40		

LDCT=low-dose computed tomography; ND=not determined; PKS=pack years; PYS=pack-years.

Table is adapted from Rossi *et al* (2005) and Yau *et al* (2007).

**Table 3:** LDCT RCT lung cancer screening trials

**Country Study name**	**LDCT**	**Control arm**	**Study design**	**Selection of participants**	**Report date**	**Publications**
The Netherlands and Belgium NELSON	8000^a^	8000^a^	LDCT *vs* no intervention	Smokers and ex-smokers with a history PKS >30 years	Recruitment completed. Report 2015	van Iersel *et al* (2007)
Denmark NELSON	2000^a^	2000^a^	LDCT *vs* no intervention	Smokers and ex-smokers with a history PKS >30 years	Recruitment underway. Report 2015	Pedersen *et al* (2002)
Italy ITALLUNG	1500	1500	LDCT *vs* no intervention	Smokers and ex-smokers PKS >30 years	Report 2005	Picozzi *et al* (2005)
DANTE	1276	1196	Chest X-ray and sputum cytology for all patients in year 1. LDCT *vs* yearly review	Smokers PKS >20 years	Report 2007	Infante *et al* (2007)
France (pilot) DepiScan	330	291	LDCT *vs* chest X-ray	Smokers 64% and former smokers (36%	Report 2006	Blanchon *et al* (2007)
United States LSS Feasibility Study	1600	1658	LDCT *vs* chest X-ray	Smokers PKS	Report 2005	Gohagan *et al* (2005)
USA NLST	26 500	26 500	LDCT *vs* chest X-ray	Current and ex-smokers PKS	Recruitment completed	http://www.cancer.gov/NSLT; Ford *et al* (2003)

LDCT=low-dose computed tomography; PKS=pack years.

a Planned recruitment.
